# Establishment of risk prediction model of postoperative pancreatic fistula after pancreatoduodenectomy: 2016 edition of definition and grading system of pancreatic fistula: a single center experience with 223 cases

**DOI:** 10.1186/s12957-021-02372-6

**Published:** 2021-08-30

**Authors:** Jun Yu, Chao-yi Ren, Jun Wang, Wei Cui, Jin-juan Zhang, Yi-jun Wang

**Affiliations:** 1 Department of Hepatobiliary Surgery, Third Central Hospital of Tianjin, Tianjin, China; 2 Tianjin Key Laboratory of Extracorporeal Life Support for Critical Diseases, Third Central Hospital of Tianjin, Tianjin, China; 3 Artificial Cell Engineering Technology Research Center of Public Health Ministry, Third Central Hospital of Tianjin, 83 Jintang Road, Tianjin, 300170 China

**Keywords:** Pancreatoduodenectomy, Pancreatic fistula, Risk prediction model

## Abstract

**Objective:**

To establish a risk prediction model for pancreatic fistula according to the pancreatic fistula standards of the 2016 edition.

**Methods:**

Clinical data from 223 patients with PD admitted to Tianjin Third Central Hospital from January 2016 to December 2020 were retrospectively analyzed. Patients were divided into modeling (January 2016 to December 2018) and validation (January 2019 to December 2020) sets according to the time of admission. The risk factors for postoperative pancreatic fistula (POPF) were screened by univariate and multivariate logistic regression analyses, and a risk prediction model for POPF was established in the modeling set. This score was tested in the validation set.

**Results:**

Logistic regression analysis showed that the main pancreatic duct index and CT value were independent risk factors according to the 2016 pancreatic fistula grading standard, based on which a risk prediction model for POPF was established. Receiver operating characteristic curve analysis showed that the area under the curve was 0.775 in the modeling set and 0.848 in the validation set.

**Conclusion:**

The main pancreatic duct index and CT value of the pancreas are closely related to the occurrence of pancreatic fistula after PD, and the established risk prediction model for pancreatic fistula has good prediction accuracy.

## Introduction

Postoperative pancreatic fistula (POPF) is the most common and serious complication after pancreatoduodenectomy (PD). In the past decade, despite improvements in surgical technology, the incidence of POPF in major central hospitals is still 5–30% [1–3]. In 2005, the International Study Group of Pancreatic Fistula (ISGPF) formulated a standard for the diagnosis and grading of POPF [4], which has been widely used in academic discussions and clinical practice. In 2016, the International Study Group on Pancreatic Surgery (ISGPS) updated the diagnosis and grading of pancreatic fistula [5], emphasizing the clinical relevance of pancreatic fistula, where the original A-level pancreatic fistula is defined as biochemical leakage but is no longer diagnosed as pancreatic fistula.

In this study, clinical data from 223 PD patients in our department at Tianjin Third Central Hospital (Tianjin, China) from January 2016 to December 2020 were reviewed, and the risk factors for pancreatic fistula after PD according to different definitions of pancreatic fistula were analyzed. A risk prediction model for pancreatic fistula after PD was established according to the new definition and grading standards of pancreatic fistula, and the accuracy of this scoring system was examined in a validation set.

## Materials and methods

### Inclusion and exclusion criteria

The inclusion criteria were as follows: patients who underwent standard procedures of PD surgery with curative intent, standard contrast-enhanced computed tomography (CT) performed less than 2 weeks before surgical resection, and no history of radiotherapy or chemotherapy. The exclusion criteria were as follows: PD combined with other organ surgery, incomplete medical records, other malignant tumors existing simultaneously, and emergency surgery for trauma. Between January 2016 and December 2020, 223 consecutive patients underwent PD in Tianjin Third Central Hospital. The patients were divided into modeling and validation sets according to the time of admission. The modeling set consisted of 124 consecutive patients who underwent PD between January 2016 and December 2018; data obtained from this group were used to evaluate the risk factors for POPF and develop a risk scoring system. External validation of the scoring system was performed by the validation set, which consisted of 99 patients who underwent PD between January 2019 and December 2020. This study was approved by the local ethics committee of Tianjin Third Central Hospital. All patients provided written informed consent and were treated in accordance with the Declaration of Helsinki.

### Operation method

Surgery was performed by five surgeons with rich pancreatic surgical experience, and the classical Child’s method was used for reconstruction of the digestive tract. Duct-to-mucosa anastomosis was conducted in 43 patients, and end-to-side anastomosis was conducted in 180 patients. A pancreatic duct drainage stent tube without a biliary stent tube was placed in all patients.

### Diagnosis and grading standard of POPF

The 2005 edition of ISGPF diagnostic standard for POPF is “when the postoperative time is ≥3 days and the amylase content in drainage fluid is more than 3 times the upper limit of the normal value of serum amylase” [4]; it is divided into grades A, B, and C according to its severity. The 2016 edition of ISGPS diagnostic standard is “when the postoperative time is ≥3 days, the amylase content in drainage fluid is more than 3 times the upper limit of the normal value of serum amylase, and it is related to the prognosis of clinical treatment” [5]. In the 2016 edition, “Grade A pancreatic fistula” in the definition of the 2005 edition has been changed to “biochemical leakage,” emphasizing that if amylase content in the drainage tube of the patient reaches the diagnostic standard without affecting the clinical treatment process and prognosis, pancreatic fistula is not considered to occur (refer to Table 1 for specific grading).

**Table 1:** Comparison of definition and grading system for POPF between 2005 and 2016

2005 ISGPF definition and grading system for POPF									
	Clinical manifestation	Special treatment^**a**^	Ultrasound or CT	Persistent drainage > 3 weeks^**%**^	Secondary operation	Death related to pancreatic fistula	Infection signs	Sepsis	Readmission
Grade A	Good	No	Negative	No	No	No	No	No	No
Grade B	Usually good	Yes/no	Negative/positive	Usually conducted	No	No	Yes	No	Yes/no
Grade C	Sickly appearance/bad	Yes	Positive	Yes	Yes	May be	Yes	Yes	Yes/no
2016 ISGPS definition and grading system for POPF									
	Increased amylase activity > 3 times upper limit institutional normal serum value	Persisting peripancreatic drainage > 3 weeks	Clinically relevant change in management of POPF^**b**^	POPF percutaneous or endoscopic specific interventions for collections	Angiographic procedures for POPF related bleeding	Reoperation for POPF	Infection signs related to POPF	Organ failure related to POPF^**c**^	Death related to POPF
Biochemical leakage	Yes	No	No	No	No	No	No	No	No
Grade B	Yes	Yes	Yes	Yes	Yes	No	Yes (no organ failure)	No	No
Grade C	Yes	Yes	Yes	Yes	Yes	Yes	Yes	Yes	Yes

*POPF* postoperative pancreatic fistula, *ISGPF* International Study Group of Pancreatic Fistula, *ISGPS* International Study Group on Pancreatic Surgery, *CT* computed tomographic scan

^**a**^Partial (peripheral) or total parenteral nutrition, antibiotics, enteral nutrition, somatostatin analog, and/or minimal invasive drainage

% With or without a drain in situ

^**b**^Prolongation of hospital or ICU stay includes use of therapeutic agents specifically employed for fistula management or its consequences (of these: somatostatin analogs, TPN/TEN, blood product transfusion, or other medications)

^**c**^Postoperative organ failure is defined as the need for re-intubation, hemodialysis, and/or inotropic agents > 24 h for respiratory, renal, or cardiac insufficiency, respectively

### Risk prediction model

In this study, two indexes were included in the prediction system for pancreatic fistula: the main pancreatic duct index and CT value of the pancreas. The main pancreatic duct index refers to the ratio of the main pancreatic duct diameter to the pancreatic thickness at the section where the pancreas is cut. The specific method used was as follows [6]. The cross section of the superior mesenteric vein converging on the liver portal vein was selected, and the diameter of the pancreatic duct at this plane was measured as the diameter of the main pancreatic duct of the patient. The longest front and back diameter of the pancreas that was perpendicular to the direction of the main pancreatic duct was selected, and the thickness of the pancreas was measured. The CT value of the pancreas refers to the CT value of the cut section of the pancreas measured on the CT plain scan image. The specific method was as follows [7]. For patients whose pancreatic duct was not expanded, the cut section was the left side of the superior mesenteric vein crossing the pancreas, with a longitudinal elliptical area. Attention was paid to avoid the splenic artery and pancreatic duct, and the CT value of this area was recorded. The CT values of three different layers of the pancreas were measured, and the average value was calculated as the CT value of the pancreas of the patient. For patients with pancreatic duct dilatation, pancreatic parenchyma occurred in the upper and lower parts of the expanded pancreatic duct. Their average CT value was calculated as the CT value of the plane, and the other calculation points were the same as those with patients without pancreatic duct dilatation.

### Statistical method

IBM SPSS 21.0 statistical software was adopted for statistical analysis. Categorical variables were compared using the chi-square test and continuous variables were compared using the *t* test or Mann–Whitney *U* test. Baseline variables that were considered clinically relevant or that showed a univariate relationship with outcome (candidate variables with a *p* value of < 0.2 on univariate analysis) was entered into multivariate logistic regression model to determine the independent risk factors for POPF. A predictive scoring system was developed using each independent risk factor, based on the regression coefficient of the logistic regression model. The receiver operating characteristic (ROC) curve was used to analyze the best sensitivity, specificity, and area under the curve (AUC) of the scoring system. Hosmer-Lemeshow goodness-of-fit test was used to evaluate the calibration degree of this system. Leave-one-out classification cross-validation experimental data. *P* < 0.05 was considered statistically significant.

## Results

### POPF in the modeling set

A total of 223 patients with PD were selected for this study, including 152 men and 71 women, aged 34–78 years (average, 61 ± 8 years); 124 and 99 patients were divided into the modeling set and validation set, respectively. Table 2 describes the whole cohort and provides a comparison between the modeling and validation sets. There was no significant difference in any characteristic between the two sets. In the modeling set, one patient underwent a secondary operation for abdominal hemorrhage caused by pancreatic fistula, and five patients died during the perioperative period (four died of abdominal infection and bleeding caused by pancreatic fistula). According to the 2005 ISGPF definition and grading system for POPF, there were 124 patients in the modeling set, of whom 61 had pancreatic fistula (23 patients with grade A, 33 had grade B, 5 had grade C), with an incidence of 49.2%. According to the 2016 ISGPS definition and grading system for POPF, there were 124 patients in the modeling set, of whom 32 had pancreatic fistula (28 patients with grade B, 4 with grade C), with an incidence of 25.8%. The new grading system reduces the original grade C pancreatic fistula to grade B pancreatic fistula, and part of grade B pancreatic fistula to biochemical leakage. In the same group, the incidence of pancreatic fistula decreased from 49.2 to 25.8% because of the change in diagnostic standard.

**Table 2:** Demographic and clinical characteristics of the modeling and validation sets

Variable	Modeling set (*n* = 124)	Validation set (*n* = 99)	*P* value
Gender (%)			
Male	85 (68.5)	67 (67.7)	0.890
Female	39 (31.5)	32 (32.3)	
Age (years)	60.9 ± 8.7	61.3 ± 6.8	0.786
Body mass index (kg/m^2^)	23.2 ± 3.1	22.5 ± 3.2	0.287
Drinking habit (%)			
Yes	23 (18.5)	26 (26.3)	0.167
No	101 (81.5)	73 (73.7)	
Smoking habit (%)			
Yes	55 (44.4)	42 (42.4)	0.773
No	69 (55.6)	57 (57.6)	
Diabetes mellitus (%)			
Yes	28 (22.6)	19 (19.2)	0.538
No	96 (77.4)	80 (80.8)	
Main pancreatic duct diameter (mm)	3.7 ± 2.5	3.6 ± 2.3	0.506
Margin pancreas thickness (mm)	15.5 ± 3.8	14.8 ± 3.3	0.210
Main pancreatic duct index	0.3 ± 0.2	0.3 ± 0.2	0.885
Portal vein invasion diagnosis^**a**^ (%)			
Yes	10 (8.1)	7 (7.1)	0.781
No	114 (91.9)	92 (92.9)	
Intra-abdominal thickness^**b**^ (mm)	70.6 ± 26.6	67.2 ± 24.3	0.352
Pancreas CT value (HU)	38.8 ± 8.5	38.3 ± 9.0	0.710
Preoperative biliary drainage (%)			
Yes	16 (12.9)	9 (9.1)	0.370
No	108 (87.1)	90 (90.9)	
Preoperative laboratory data			
White blood cell count (10^9^/L)	6.1 ± 1.9	5.9 ± 1.8	0.450
Platelet count (10^9^/L)	233.4 ± 69.6	241.0 ± 63.7	0.275
Albumin (g/L)	39.4 ± 4.2	38.8 ± 3.9	0.551
Total bilirubin (μmol /L)	150.5 ± 130.5	141.7 ± 110.6	0.691
Amylase (IU/L), median (IQR)	25 (16–42)	27 (19.8–39.95)	0.621
CA19-9 (U/mL), median (IQR)	82 (35.1–276.8)	101 (27.5–381.3)	0.708
Pancreaticojejunostomy (%)			
Duct-to-mucosa	26 (21.0)	13 (13.1)	0.126
Dunking method	98 (79.0)	86 (86.9)	
Pancreatic cancer^c^ (%)			
Yes	29 (23.4)	25 (25.3)	0.747
No	95 (76.6)	74 (74.7)	
Postoperative pancreatic fistula (%)			
2005 ISGPF edition			
Yes	61 (49.2)	43 (43.4)	0.392
No	63 (50.8)	56 (56.6)	
2016 ISGPS edition			
Yes	32 (25.8)	23 (23.2)	0.658
No	92 (74.2)	76 (76.8)	
Surgery-related death (%)			
Yes	5 (4.0)	3 (3.0)	0.970
No	119 (96.0)	96 (97.0)	

*CT* computed tomographic scan, *IQR* interquartile range, *CA19-9* carbohydrate antigen 19-9, *ISGPF* International Study Group of Pancreatic Fistula, *ISGPS* International Study Group on Pancreatic Surgery

^**a**^The tumors that were attached, compressed, or obviously involved in the portal and/or superior mesenteric veins on CT

^**b**^Measured as the distance from the internal face of rectus abdominis (linea alba) to the rear wall of the aorta at the level of the umbilicus

^c^124 patients in the modeling set: pancreatic cancer 29, cholangiocarcinoma 28, ampulla carcinoma 52, duodenal cancer 10, duodenal papillitis 1, pancreatitis 1, duodenal papilloma 1, pancreatic head neuroendocrine tumor 1, cholangitis 1; 99 patients in the validation set: pancreatic cancer 25, cholangiocarcinoma 15, ampulla carcinoma 46, duodenal cancer 7, pancreatitis 1, intraductal papillary mucinous carcinoma of the pancreas (IPMN) 1, pancreatic serous cystadenoma 2, solid pseudopapillary tumor of pancreas 1, pancreatic mucinous cystadenoma 1

### Risk factors related to pancreatic fistula in the modeling set

#### 2005 ISGPF definition and grading system for POPF

The results of univariate analysis showed that the main pancreatic duct diameter, main pancreatic duct index, portal vein invasion diagnosis, intra-abdominal thickness, preoperative biliary drainage, pancreatic cancer diagnosis, margin pancreas thickness, pancreas CT value, and preoperative serum amylase level were related to POPF (Table 3). Variables that were considered clinically relevant or that showed a univariate relationship with outcome (candidate variables with a *p* value < 0.2 on univariate analysis) were entered into multivariate logistic regression model to determine independent risk factors for POPF. The results showed that the main pancreatic duct diameter, main pancreatic duct index, pancreatic cancer diagnosis, and pancreas CT value were independent risk factors for POPF (Table 3).

**Table 3:** Univariate and multivariate analysis results of risk factors related to POPF after PD (2005 ISGPF edition)

Variable	Univariate analysis			Multivariate analysis		
	Odds ratio	95% CI	*P* value	Odds ratio	95% CI	*P* value
Sex (male / female)	1.389	0.648–2.977	0.398			
Age (**≥** 65 years/<65 years)	0.837	0.392–1.790	0.647			
Body mass index (**≥**23/<23)	1.597	0.767–3.328	0.210			
Drinking habit (yes/no)	0.935	0.378–2.314	0.884			
Smoking habit (yes/no)	1.131	0.557–2.299	0.733			
Diabetes mellitus (yes/no)	0.595	0.253–1.403	0.233			
Main pancreatic duct diameter (< 3 mm/≥ 3 mm)	6.464	2.956–14.136	0.000	4.210	1.399–12.669	0.011
Main pancreatic duct index (<0.21/≥ 0.21)	5.532	2.563–11.943	0.000	4.561	1.480–14.059	0.008
Portal vein invasion^**a**^ (no/yes)	10.000	1.226–81.534	0.024			
Intra-abdominal thickness^**b**^ (≥ 69 mm/< 69 mm)	3.083	1.482–6.412	0.002			
Preoperative biliary drainage (no/yes)	3.612	1.095–11.913	0.027			
Pancreatic cancer (no/yes)	2.687	1.110–6.507	0.025	5.517	1.546–19.688	0.009
Pancreatic resection margin thickness (**≥** 15 mm/< 15 mm)	2.200	1.058–4.573	0.032			
Pancreas CT value (< 39 HU/**≥** 39 HU)	2.681	1.297–5.541	0.007	8.981	2.934–27.490	0.000
White blood cell count (**≥** 9.5×10^9^/L/< 9.5×10^9^/L)	0.678	0.109–4.205	0.675			
Albumin (< 40 g/L/**≥** 40 g/L)	1.395	0.686–2.838	0.358			
Total bilirubin (**≥** 20 μmol/L/<20 μmol/L)	1.857	0.717–4.813	0.199			
Amylase (< 110 IU/L/**≥** 110 IU/L)	0.206	0.042–0.999	0.033			
CA19-9 (< 39 U/mL/**≥** 39 U/mL)	2.096	0.929–4.729	0.112			
Pancreaticojejunostomy (dunking method/duct-to-mucosa)	0.960	0.404–2.280	0.926			

Continuous variables were classified into two groups as follows: The thresholds of body mass index, main pancreatic duct diameter, main pancreatic duct index, intra-abdominal thickness, pancreatic resection margin thickness, and pancreas CT value were determined based on the median value of each parameter. All laboratory data were divided based on the upper or lower limit of normal range of each parameter

*POPF* postoperative pancreatic fistula, *PD* pancreatoduodenectomy, *ISGPF* International Study Group of Pancreatic Fistula, *CI* confidence

interval, *CT* computed tomographic scan, *CA19-9* carbohydrate antigen 19-9

^**a**^The tumors that were not attached, compressed, or obviously involved the portal and/or superior mesenteric veins on CT

^**b**^Measured as the distance from the internal face of rectus abdominis (linea alba) to the rear wall of the aorta at the level of the umbilicus

#### 2016 ISGPS definition and grading system of POPF

The results of univariate analysis showed that the main pancreatic duct diameter, main pancreatic duct index, intra-abdominal thickness, margin pancreas thickness, and pancreas CT value were related to POPF (Table 4). Variables that were considered clinically relevant or that showed a univariate relationship with outcome (candidate variables with a p value <0.2 on univariate analysis) were entered into multivariate logistic regression model to determine independent risk factors for POPF. The results showed that the main pancreatic duct index and pancreas CT value were independent risk factors for POPF (Table 4).

**Table 4:** Univariate and multivariate analysis results of risk factors related to POPF after PD (2016 ISGPS edition)

Variable	Univariate analysis			Multivariate analysis		
	Odds ratio	95% CI	*P* value	Odds ratio	95% CI	*P* value
Sex (male/female)	1.425	0.609–3.330	0.413			
Age (**≥** 65 years/< 65 years)	1.200	0.532–2.706	0.660			
Body mass index (**≥** 23/< 23)	1.864	0.854–4.068	0.116			
Drinking habit (yes/no)	0.988	0.369–2.642	0.981			
Smoking habit (yes/no)	1.192	0.554–2.566	0.653			
Diabetes mellitus (yes/no)	0.545	0.201–1.479	0.229			
Main pancreatic duct diameter (< 3 mm/≥ 3 mm)	3.126	1.330–7.351	0.007			
Main pancreatic duct index (< 0.21/≥ 0.21)	4.263	1.731–10.500	0.001	4.912	1.931–12.493	0.001
Portal vein invasion* (no/yes)	1.500	1.317–1.708	0.067			
Intra-abdominal thickness** (≥ 69 mm/< 69 mm)	3.284	1.406–7.669	0.005			
Preoperative biliary drainage (no/yes)	2.383	0.856–7.205	0.087			
Pancreatic cancer (no/yes)	0.391	0.137–1.121	0.074			
Pancreatic resection margin thickness (**≥** 15 mm/< 15 mm)	3.134	1.233–7.966	0.014			
Pancreas CT value (< 39 HU/**≥** 39 HU)	3.185	1.515–6.695	0.002	2.503	1.033–6.064	0.042
White blood cell count (**≥** 9.5×10^9^/L/< 9.5×10^9^/L)	1.368	1.227–1.525	0.410			
Albumin (< 40 g/L/**≥** 40 g/L)	1.828	0.792–4.218	0.155			
Total bilirubin (**≥** 20 μmol/L/< 20 μmol /L)	2.087	0.653–6.668	0.207			
Amylase (< 110 IU/L/**≥** 110 IU/L)	1.824	0.372–8.944	0.692			
CA19-9 (< 39 U/mL/**≥** 39 U/mL)	1.812	0.763–4.303	0.175			
Pancreaticojejunostomy (dunking method/duct-to-mucosa)	0.794	0.317–1.986	0.621			

Continuous variables were classified into two groups as follows: The thresholds of body mass index, main pancreatic duct diameter, main pancreatic duct index, intra-abdominal thickness, pancreatic resection margin thickness, and pancreas CT value were determined based on the median value of each parameter. All laboratory data were divided based on the upper or lower limit of normal range of each parameter

*POPF* postoperative pancreatic fistula, *PD* pancreatoduodenectomy, *ISGPF* International Study Group of Pancreatic Fistula, *CI* confidence interval, *CT* computed tomographic scan, *CA19-9* carbohydrate antigen 19-9

^**a**^The tumors that were not attached, compressed, or obviously involved the portal and/or superior mesenteric veins on CT

^**b**^Measured as the distance from the internal face of rectus abdominis (linea alba) to the rear wall of the aorta at the level of the umbilicus

### Establishing a risk prediction model for POPF in the modeling set

The logistic regression probability equation, i.e., risk probability model for POPF after PD, was obtained according to the multivariate analysis results in the 2016 ISGPS definition and grading system for POPF (Table 5).

**Table 5:** Predictive scoring system for POPF according to the logistic regression analysis results in the modeling set (2016 ISGPS edition)

Variable	Coefficient	Odds ratio	95% CI	*P* value
Main pancreatic duct index	−7.599	0.001	0.000–0.035	0.000
Pancreas CT value	−0.064	0.938	0.885–0.993	0.028
Constant	3.111	22.443		0.018

Continuous variables were directly included in the logistic regression analysis as the independent variables to establish risk prediction model

*POPF* postoperative pancreatic fistula, *ISGPF* International Study Group of Pancreatic Fistula, *CI* confidence interval, *CT* computed tomographic scan

*P* = 1/[1 + e − (3.111 − 7.599 × main pancreatic duct index − 0.064 × pancreas CT value)]

### Diagnostic value of the risk prediction model

**Fig. 1:**
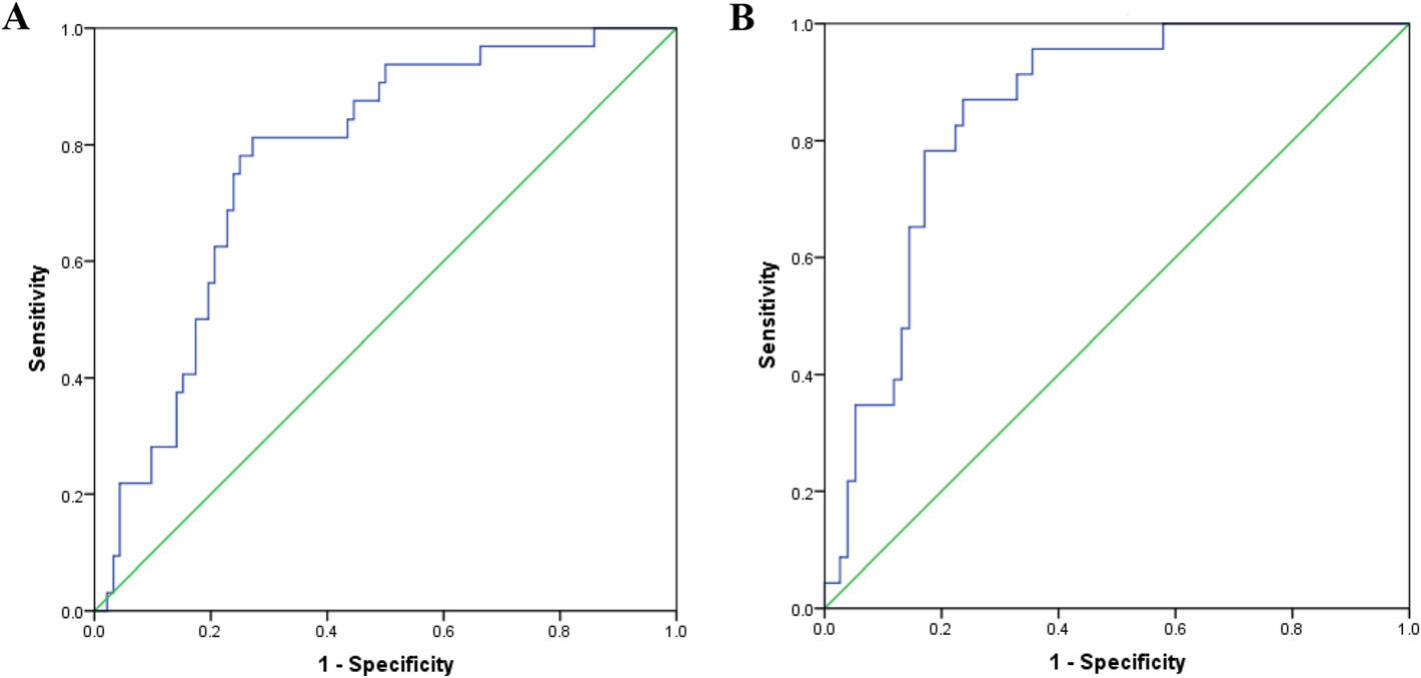
Receiver operating characteristic (ROC) curve of predictive model in modeling set (**A**) and validation set (**B**). The area under the ROC curve was 0.775 and 0.848, respectively, for modeling and validation set

ROC curve analysis of the prediction model showed that when the cut-off value (*P* value) was 30%, the sensitivity of the ROC curve was 81.3%, the specificity was 72.8%, and the AUC was 0.775 (95% CI, 0.687–0.862; Fig. 1A). The patient was considered to be at high risk for POPF when the *P* value was ≥ 30%. The greater the *P* value, the higher the risk for POPF.

### Validation of the risk prediction model

**Fig. 2:**
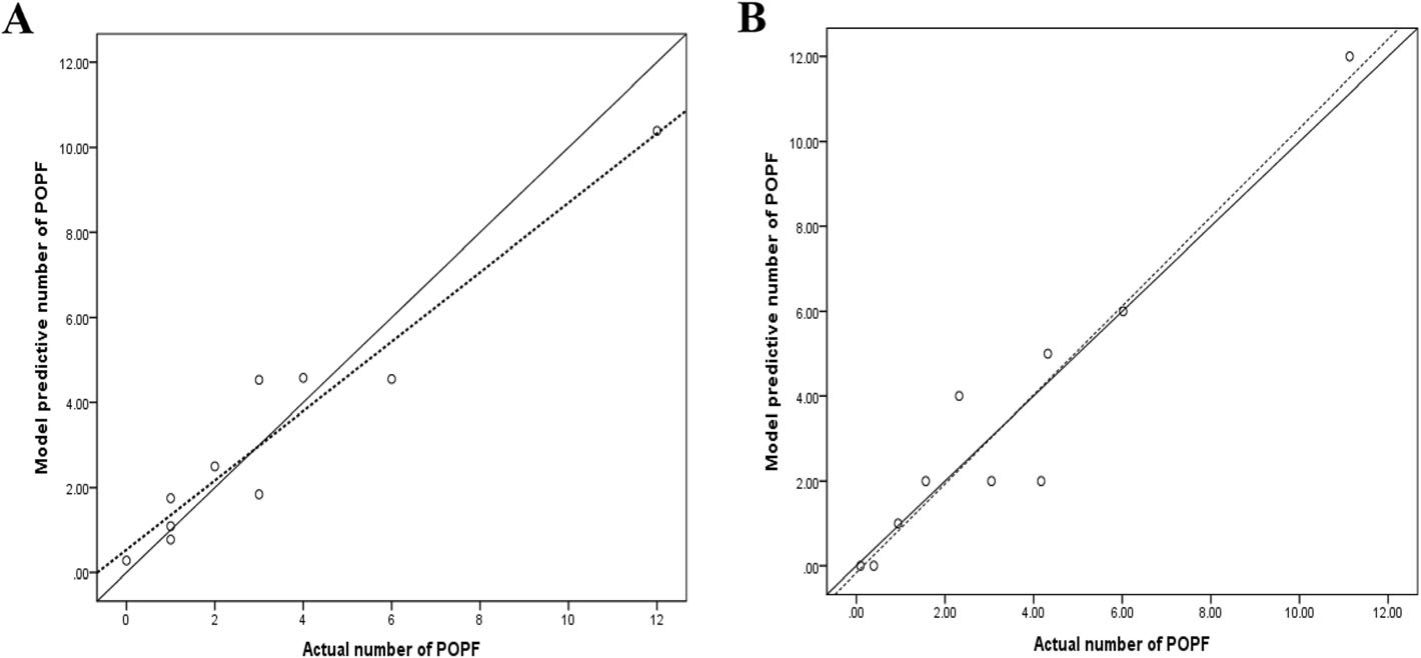
Drawing of the calibration chart to visually evaluate the predictive model. Chart A for modeling set and chart B for validation set. The solid lines indicate the standard curve; the dashed lines indicate the calibration curve; and each point represents a group. The calibration curve is close to the standard curve, suggesting that the prediction model has a good calibration ability. POPF, postoperative pancreatic fistula

To validate the risk prediction model, it was applied to the validation set. The area under the ROC curve was found to be 0.848 (95% CI, 0.770–0.926; Fig. 1B). The Hosmer-Lemeshow goodness-of-fit test was used to further evaluate the performance of this model. The results showed *χ*^2^ = 8.390, *P* = 0.396 in the modeling set and *χ*^2^ = 4.474, *P* = 0.812 in the validation set. These data indicated that the difference between the predicted value of the model and the actual observed value was not statistically significant, and the prediction model had good calibration ability. The results were visualized by drawing a calibration chart. As Fig. 2 shows, we sorted the prediction probabilities of each research object from small to large and divided them into 10 groups according to deciles. The actual observation values and the predicted values of the model in each group were expressed in the form of coordinate points, so that the difference was visually displayed for each group. Leave-one-out classification to cross-validate the experimental data. The accuracy of the initial experiment and cross-validation was 76% and 75%, respectively.

## Discussion

In 2016, the ISGPS (formerly known as ISGPF) revised the definition and grading standard for POPF, changing the “Grade A pancreatic fistula” in the 2005 edition to “biochemical leakage,” and “biochemical leakage” is no longer considered to be a kind of actual pancreatic fistula. The diagnosis of grade B pancreatic fistula needs to be clinically related and affect the postoperative process. On the basis of “biochemical leakage,” any of the following situations can be found, such as continuous drainage of abdominal cavity for more than 3 weeks, change of treatment measures for clinically related pancreatic fistula, percutaneous or endoscopic drainage, angiographic intervention in the treatment of bleeding, and infection signs without organ failure, then grade B fistula can be diagnosed. If it is needed to conduct secondary operation for grade B pancreatic fistula, and single or multiple organ failure or death occurs, it will be upgraded to grade C.

In this study, patients with pancreatic fistula were included according to the old standard in the 2005 edition and the revised standard in the 2016 edition, and the influence of new and old editions on the risk factors for POPF after PD were compared. According to the new edition of the pancreatic fistula standard, the incidence of pancreatic fistula in this study decreased from 49.2 to 25.8%, in line with clinical practice. Univariate analysis showed that there were nine risk factors in the old edition of pancreatic fistula standard including main pancreatic duct diameter, main pancreatic duct index, portal vein invasion diagnosis, intra-abdominal thickness, preoperative biliary drainage, pancreatic cancer diagnosis, margin pancreatic thickness, pancreas CT value, and preoperative serum amylase level. The new edition of pancreatic fistula standard was reduced to five including main pancreatic duct diameter, main pancreatic duct index, intra-abdominal thickness, margin pancreatic thickness, and pancreas CT value. It was suggested that these five risk factors were more closely related to clinical pancreatic fistula. Multivariate analysis showed that there were four independent risk factors in the old edition of pancreatic fistula standard including the main pancreatic duct diameter, main pancreatic duct index, pancreatic cancer diagnosis, and pancreas CT value. The new edition of pancreatic fistula standard was reduced to two, including the main pancreatic duct index and pancreas CT value. The main pancreatic duct index and pancreas CT value could be obtained before operation. Based on this, a mathematical model for predicting pancreatic fistula was established. The calculated result of the model was the POPF probability of patients. When the calculated value was more than 30%, the patient was considered high risk for POPF. The greater the calculated value, the higher the risk of POPF.

To improve the accuracy of pancreatic fistula risk prediction, many risk factors have been combined to establish a pancreatic fistula prediction system by domestic and foreign scholars. At present, the main pancreatic duct diameter and the pancreas texture are generally recognized as risk factors related to the pancreatic fistula [8–14]. Other factors include age, gender, main pancreatic duct index, body mass index, intra-abdominal thickness, pathological diagnosis, intraoperative blood loss, preoperative blood amylase, operative drainage, portal vein invasion, and reconstruction methods, all of which have been reported to be related to pancreatic fistula [12, 15–23]. According to the definition and grading standards for pancreatic fistula in the new and old editions, the risk factors for pancreatic fistula were screened out in the study, consistent with the literature. Among them, the main pancreatic duct index and pancreatic CT value were both independent risk factors for POPF after PD in the old and new editions. The main pancreatic duct index was the ratio of the main pancreatic duct diameter to the pancreatic thickness. It was proposed by Akamatsu et al. [18], and is the strongest independent predictor of POPF. This index can better predict the occurrence of pancreatic fistula than the pancreatic duct diameter can do alone [16, 24, 25]. There are two reasons why the pancreas CT value replaces the soft and hard texture of pancreas in this study. First, there is no universally recognized standard for the soft and hard texture of pancreas, which is mainly judged by the operator’s touch during operation, and the subjective factors are too strong to be quantified. Second, the literature has proven that the pancreatic texture is related to the pancreas CT value [7, 26–28]; the higher the CT value of the pancreas, the higher the density of the pancreatic tissue, the more severe the degree of pancreatic fibrosis, and the lower the risk of pancreatic fistula. In this study, it was also believed that the pancreas CT value can reflect the pancreas texture. Among the 124 patients, the pancreas CT value of patients with pancreatic fistula was 36.36 ± 6.49 in the 2005 edition and 36.16 ± 7.29 in the 2016 edition. The pancreas CT value of non-pancreatic fistula patients was 41.10 ± 9.61 in the 2005 edition and 39.67 ± 8.78 in the 2016 edition. The difference was statistically significant.

In 2011, the Japanese scholar, Yamamoto et al. [29] established a preoperative pancreatic fistula prediction system based on sex, pancreatic cancer diagnosis, main pancreatic duct index, portal vein invasion, and intra-abdominal thickness. The results showed that the prediction accuracy was high and verified by many domestic medical centers. In 2014, Roberts et al. [30] from the UK established a pancreatic fistula prediction system based on body mass index and main pancreatic duct diameter. The prediction accuracy was verified in the Center. In this study, the prediction model was established with the prediction parameters including the main pancreatic duct index and CT value of the pancreas. With the reduction of the main pancreatic duct index and CT value of the pancreas, the risk of POPF increased. The results of this study showed that the sensitivity and specificity of the prediction system were 81.3% and 72.8%, respectively. It indicated that the incidence of POPF of 124 patients receiving PD was accurately predicted. By comparing the weights of different parameters, in this study, the mathematical model was established, the probability of POPF was predicted before the surgery, and patients at a high risk of POPF were identified, which provided a reference for the intraoperative decision-making and postoperative prevention and control of surgeons.

According to the new definition and grading standard of pancreatic fistula, the prediction model of POPF after PD was established in this study. The prediction parameters can be obtained by CT before operation. The clinical operation is simple, objective, and quantitative; and the repeatability is strong. The model is of clinical value in predicting the risk of POPF before surgery. However, this study was a single-center, small sample size, and retrospective study. The relationship between preoperative CT parameters and pancreatic fistula, as well as prospective study with large samples verifies the prediction model is the future research direction.

## Conclusions

The established risk prediction model for pancreatic fistula has good prediction accuracy. This model is of clinical value in predicting the risk of POPF before surgery. Patients at a high risk of POPF were identified, which provided a reference for the intraoperative decision-making and postoperative prevention and control of surgeons. Prospective studies with large samples are needed in the future to verify the prediction model.

## Data Availability

The datasets used and/or analyzed during the current study are available from the corresponding author on reasonable request.
